# Supplementary material for: Modeling glioblastoma heterogeneity as a dynamic network of cell states
Source: Mol Syst Biol. 2021 Sep 16;17(9):e10105. doi: 10.15252/msb.202010105 (PMC8444284; doi:10.15252/msb.202010105)
Supplement: Supplementary file 6 — Source Data for Figure 5 [file MSB-17-e10105-s004.zip › Figure5A_sourcedata/GSEA_3017/nosigdb_stateB.GseaPreranked.1622795021725/RICHARDS_NOWAKOWSKI_SCIENCE_2017_EN_PFC2_UPREG.html]

Details for gene set RICHARDS\_NOWAKOWSKI\_SCIENCE\_2017\_EN\_PFC2\_UPREG[GSEA]

|  || Dataset | state43017 |
| Phenotype | NoPhenotypeAvailable |
| Upregulated in class | na\_neg |
| GeneSet | RICHARDS\_NOWAKOWSKI\_SCIENCE\_2017\_EN\_PFC2\_UPREG |
| Enrichment Score (ES) | -0.20959967 |
| Normalized Enrichment Score (NES) | -2.1368084 |
| Nominal p-value | 0.0 |
| FDR q-value | 0.007043249 |
| FWER p-Value | 0.018 |
Table: GSEA Results Summary

  

Fig 1: Enrichment plot: RICHARDS\_NOWAKOWSKI\_SCIENCE\_2017\_EN\_PFC2\_UPREG      
 Profile of the Running ES Score & Positions of GeneSet Members on the Rank Ordered List

  

| PROBE | GENE SYMBOL | GENE\_TITLE | RANK IN GENE LIST | RANK METRIC SCORE | RUNNING ES | CORE ENRICHMENT || 1 | LRRN3 |  |  | 25 | 0.704 | -0.0065 | No |
| 2 | LIMCH1 |  |  | 88 | 0.552 | -0.0732 | No |
| 3 | CSRP2 |  |  | 96 | 0.539 | -0.0605 | No |
| 4 | CMC2 |  |  | 105 | 0.516 | -0.0502 | No |
| 5 | CEP78 |  |  | 161 | 0.451 | -0.1110 | No |
| 6 | NCAM1 |  |  | 178 | 0.436 | -0.1158 | No |
| 7 | CDKN2D |  |  | 190 | 0.427 | -0.1137 | No |
| 8 | EPS8 |  |  | 257 | 0.371 | -0.1938 | Yes |
| 9 | TUBB |  |  | 263 | 0.368 | -0.1855 | Yes |
| 10 | ASRGL1 |  |  | 266 | 0.366 | -0.1729 | Yes |
| 11 | TUBA1C |  |  | 270 | 0.362 | -0.1619 | Yes |
| 12 | SDK1 |  |  | 282 | 0.358 | -0.1627 | Yes |
| 13 | TENM1 |  |  | 291 | 0.354 | -0.1593 | Yes |
| 14 | DCBLD2 |  |  | 294 | 0.351 | -0.1473 | Yes |
| 15 | TUBB2A |  |  | 296 | 0.351 | -0.1339 | Yes |
| 16 | DOK5 |  |  | 302 | 0.346 | -0.1264 | Yes |
| 17 | BCAP29 |  |  | 308 | 0.343 | -0.1191 | Yes |
| 18 | OLFM1 |  |  | 325 | 0.337 | -0.1281 | Yes |
| 19 | PRSS23 |  |  | 329 | 0.334 | -0.1183 | Yes |
| 20 | AIG1 |  |  | 339 | 0.330 | -0.1174 | Yes |
| 21 | GRIA4 |  |  | 358 | 0.322 | -0.1299 | Yes |
| 22 | RCAN1 |  |  | 361 | 0.321 | -0.1192 | Yes |
| 23 | SOGA1 |  |  | 378 | 0.314 | -0.1291 | Yes |
| 24 | TTYH3 |  |  | 396 | 0.309 | -0.1407 | Yes |
| 25 | CPNE8 |  |  | 397 | 0.309 | -0.1275 | Yes |
| 26 | GCLM |  |  | 401 | 0.308 | -0.1189 | Yes |
| 27 | MEIS2 |  |  | 405 | 0.307 | -0.1102 | Yes |
| 28 | PPP1R14C |  |  | 407 | 0.305 | -0.0987 | Yes |
| 29 | RGS17 |  |  | 416 | 0.300 | -0.0976 | Yes |
| 30 | SEMA3A |  |  | 460 | 0.290 | -0.1478 | Yes |
| 31 | IDS |  |  | 465 | 0.289 | -0.1413 | Yes |
| 32 | APLP1 |  |  | 480 | 0.285 | -0.1496 | Yes |
| 33 | TTLL7 |  |  | 497 | 0.281 | -0.1609 | Yes |
| 34 | COTL1 |  |  | 499 | 0.281 | -0.1504 | Yes |
| 35 | CAMK2N1 |  |  | 505 | 0.280 | -0.1458 | Yes |
| 36 | EML1 |  |  | 515 | 0.278 | -0.1471 | Yes |
| 37 | KIFAP3 |  |  | 518 | 0.278 | -0.1382 | Yes |
| 38 | CDC27 |  |  | 520 | 0.278 | -0.1278 | Yes |
| 39 | TSPAN13 |  |  | 528 | 0.276 | -0.1263 | Yes |
| 40 | SMARCA2 |  |  | 530 | 0.276 | -0.1160 | Yes |
| 41 | NAV3 |  |  | 534 | 0.275 | -0.1087 | Yes |
| 42 | RORB |  |  | 555 | 0.271 | -0.1263 | Yes |
| 43 | PBX1 |  |  | 567 | 0.270 | -0.1308 | Yes |
| 44 | RTN1 |  |  | 573 | 0.269 | -0.1267 | Yes |
| 45 | KHDRBS3 |  |  | 599 | 0.263 | -0.1519 | Yes |
| 46 | PRKAR2B |  |  | 611 | 0.262 | -0.1568 | Yes |
| 47 | LMO4 |  |  | 615 | 0.261 | -0.1501 | Yes |
| 48 | ICA1L |  |  | 621 | 0.259 | -0.1463 | Yes |
| 49 | CHCHD3 |  |  | 628 | 0.259 | -0.1441 | Yes |
| 50 | MPRIP |  |  | 631 | 0.258 | -0.1360 | Yes |
| 51 | NRCAM |  |  | 633 | 0.258 | -0.1266 | Yes |
| 52 | DPYD |  |  | 634 | 0.258 | -0.1156 | Yes |
| 53 | PRKACB |  |  | 636 | 0.257 | -0.1062 | Yes |
| 54 | MAGI3 |  |  | 648 | 0.256 | -0.1113 | Yes |
| 55 | ATG5 |  |  | 659 | 0.255 | -0.1150 | Yes |
| 56 | LONRF2 |  |  | 677 | 0.251 | -0.1291 | Yes |
| 57 | HMGCR |  |  | 683 | -0.257 | -0.1254 | Yes |
| 58 | TUBA1A |  |  | 686 | -0.263 | -0.1172 | Yes |
| 59 | SCD |  |  | 688 | -0.263 | -0.1075 | Yes |
| 60 | DCX |  |  | 689 | -0.264 | -0.0963 | Yes |
| 61 | MSMO1 |  |  | 691 | -0.267 | -0.0864 | Yes |
| 62 | RPL37 |  |  | 696 | -0.274 | -0.0806 | Yes |
| 63 | GDI2 |  |  | 697 | -0.274 | -0.0690 | Yes |
| 64 | PCDH10 |  |  | 713 | -0.305 | -0.0778 | Yes |
| 65 | MARCKSL1 |  |  | 716 | -0.311 | -0.0676 | Yes |
| 66 | NDUFB8 |  |  | 718 | -0.323 | -0.0553 | Yes |
| 67 | IDI1 |  |  | 734 | -0.397 | -0.0603 | Yes |
| 68 | SQLE |  |  | 735 | -0.405 | -0.0431 | Yes |
| 69 | HMGCS1 |  |  | 739 | -0.418 | -0.0297 | Yes |
| 70 | PPA1 |  |  | 742 | -0.424 | -0.0146 | Yes |
| 71 | GAP43 |  |  | 743 | -0.428 | 0.0035 | Yes |
| 72 | MLLT11 |  |  | 744 | -0.430 | 0.0218 | Yes |
Table: GSEA details [plain text format]

  

Fig 2: RICHARDS\_NOWAKOWSKI\_SCIENCE\_2017\_EN\_PFC2\_UPREG: Random ES distribution      
 Gene set null distribution of ES for **RICHARDS\_NOWAKOWSKI\_SCIENCE\_2017\_EN\_PFC2\_UPREG**
